# A20 (TNFAIP3) Alleviates CVB3-Induced Myocarditis via Inhibiting NF-κB Signaling

**DOI:** 10.1371/journal.pone.0046515

**Published:** 2012-09-28

**Authors:** Jun Gui, Yan Yue, Ruizhen Chen, Wei Xu, Sidong Xiong

**Affiliations:** 1 Institute for Immunobiology, Shanghai Medical College, Fudan University, Shanghai, People’s Republic of China; 2 Institutes of Biology and Medical Sciences, Soochow University, Suzhou, People’s Republic of China; 3 Key Laboratory of Viral Heart Diseases, Shanghai Institute of Cardiovascular Diseases, Zhongshan Hospital, Fudan University, Shanghai, People’s Republic of China; UAE University, United Arab Emirates

## Abstract

**Background:**

Viral myocarditis, which is most prevalently caused by Coxsackievirus B3 (CVB3) infection, is a serious clinical condition characterized by cardiac inflammation. However, efficient therapies targeting inflammation are still lacking and much needed. A20, also known as tumor necrosis factor alpha induced protein 3 (TNFAIP3) is a key negative regulator of inflammation. But whether A20 may affect cardiac inflammation during acute viral myocarditis remains to be elucidated. The aim of this study was to investigate the potential protective effect of A20 on CVB3-induced myocarditis.

**Methodology/Principal Findings:**

Mice were intraperitoneally inoculated with CVB3 to establish acute viral myocarditis model. We found that the expression of pro-inflammatory cytokines, including tumor necrosis factor-α (TNF-α), interleukin (IL)-1β, IL-6 and monocyte chemotactic protein-1 (MCP-1) were markedly and persistently increased during the progression of CVB3-induced myocarditis, and positively correlated with the disease severity. Notably, intravenous injection *in vivo* with adenovirus expressed A20 (Ad-A20) remarkably reduced CVB3-induced pro-inflammatory cytokines production and alleviated the severity of myocarditis. Further, we observed that nuclear factor-kappaB (NF-κB) signaling which mediates inflammatory response was significantly inhibited in CVB3-infected mice with Ad-A20 treatment. Finally, we revealed that A20 was required to inhibit CVB3-induced NF-κB signaling by restricting TNF receptor associated factor 6 (TRAF6) ubiquitylation.

**Conclusion/Significance:**

This study demonstrates the protective role of A20 against CVB3-induced myocarditis, which may provide a new therapeutic strategy for the treatment of viral myocarditis.

## Introduction

Viral myocarditis is a principal cause of heart failure in young adults and often progresses to chronic myocarditis, dilated cardiomyopathy, and congestive heart failure. Coxsackievirus B3 (CVB3) is believed to be the most common causative agent in human myocarditis, and the same virus strain induced similar inflammatory heart disease in genetically susceptible strains of mice [1]–[3]. Despite decades of extensive effort, the pathogenesis of viral myocarditis is still not fully understood and there is no effective therapy for this disease so far. Experimental studies have found that although CVB3 can directly destroy myocardium [4]–[6], the overwhelming inflammatory response is primarily responsible for myocyte damage [7]–[9]. Clinical studies have also found increased levels of circulating tumor necrosis factor-α (TNF-α), interleukin (IL)-1β, IL-6 and other pro-inflammatory cytokines in patients with myocarditis [10], [11]. And certain immunosuppressive drugs are used to control inflammation in clinical treatment [12]. Therefore modulation of inflammatory response considers as a potential therapeutic strategy for viral myocarditis.

In fact, several approaches have been reported to modulate inflammatory response for treating viral myocarditis in mice. For instance, studies showed that direct blockade of inflammatory cytokines including TNF-α, monocyte chemotactic protein-1 (MCP-1) and IL-17 by using neutralizing antibodies (Abs) could attenuate myocardial inflammation and resulted in disease remission [13]–[15]. Besides, it has been found that T cell immune response mediate cytokine pattern present in viral myocarditis. Both our and other research groups demonstrated that modulation of CD4^+^ Th immune response to a Th2 profile and activation of regulatory T cells (Tregs) might prevent CVB3-induced cardiac inflammation [16]–[19]. Our previous work also found that transfer of M2 macrophages into susceptible male mice could alleviate myocardial inflammation by modulating local cytokine profile [20]. However, efficient therapies targeting inflammation are still needed further development and exploiting new therapeutic strategies is much necessary.

A20, also known as TNF-α induced protein 3 (TNFAIP3) is a cytoplasmic protein that plays a key role in the negative regulation of inflammatory response [21], [22]. Experimental studies have indicated its critical role for preventing inflammation *in vivo*. A20 deficient mice spontaneously develop severe inflammation and die prematurely due to severe multi-organ inflammation and cachexia [23]. And the protective role of A20 has been reported in several inflammatory diseases, including bronchial asthma, rheumatoid arthritis and atherosclerosis [24]–[26]. However, whether A20 may affect cardiac inflammation during viral myocarditis is still unclear. Therefore, this study was performed to examine the potential protective effect of A20 on CVB3-induced myocarditis.

To address this issue, a recombinant adenoviral vector expressing A20 (Ad-A20) was constructed. Mice were intravenously injected with adenovirus Ad-A20 2 days before CVB3 inoculation. Inflammatory cytokine expression profiles in cardiac tissues of CVB3 infected mice with Ad-A20 treatment were analyzed. The therapeutic effect of this strategy on viral myocarditis was assessed carefully and its possible mechanism involved was explored.

## Materials and Methods

### Ethics Statement

All experiments carried out in this study were strictly performed in a manner to minimize suffering of laboratory mice. All animal procedures were performed according to the Guide for the Care and Use of Medical Laboratory Animals (Ministry of Health, P.R. China, 1998) and with the ethical approval of the Shanghai Medical Laboratory Animal Care and Use Committee (Permit number: SYXK 2009-0036) as well as the Ethical Committee of Fudan University (Permit number: 2009016).

### Mice and Virus

Specific pathogen free Male BALB/c mice (H-2^d^), 6 weeks of age were purchased from Shanghai Experimental Animal Centre of Chinese Academy of Sciences. CVB3 (Nancy strain) was maintained by passage through HeLa cells (ATCC number: CCL-2). Viral titer was routinely determined prior to infection by a 50% tissue culture infectious dose (TCID_50_) assay of HeLa cell monolayer.

### Preparation of Adenovirus

Adenoviruses encoding A20 (Ad-A20) and the control (Ad-LacZ) were created using the Virapower adenovirus expression system according to the manufacturer’s instructions (Invitrogen). Mouse A20 cDNA fragment from lipopolysaccharide (LPS) stimulated RAW264.7 cells was amplified using primers 5′-T*GGATCC*CGCGGCCCCAAGAGGCCTT.

GTCGAG-3′ and 5′-T*CTCGAG*TGTCAATGTGTTCGCACTTAGCCATACATC-3' by reverse transcription polymerase chain reaction (RT-PCR). The resulting PCR product was digested with restriction enzyme *Bam*H I and *Xho* I, then inserted into pENTR^3C^ (Invitrogen) to yield entry vector pENTR-A20.The insert and junctions were sequenced to verify the absence of mutations. Site specific recombination between pENTR-A20 and the adenoviral destination vector (pAd/CMV/V5-DEST) were established with LR clonase II (Invitrogen). Digest the recombinant plasmid pAd-CMV-A20 and the control pAd-CMV-LacZ with *Pac* I. Transfect 1 µg of *Pac* I-digested pAd-DEST expression plasmid into 293A cells (Invitrogen). Harvest culture supernatants of 293A cells when visible regions of cytopathic effect were observed. This stock was used to infect 293A cells to generate a higher titer viral stock. Adenovirus using *in vivo* were purified by cesium chloride banding. Virus titers were determined by a plaque assay using serial dilution.

### CVB3 Infection and Adenoviral Delivery in Mice

Six mice in each group were infected by an intraperitoneal injection with10^3^ TCID_50_ CVB3 at day 0. To examine the therapeutic effects of A20, the mice received intravenous injection of 50 µl of adenovirus Ad-A20 or the control Ad-LacZ (3×10^9^ plaque forming units, pfu) 2 days before CVB3 infection. Infected mice receiving saline treatment were used as control.

### Tissue Histopathology and Myocarditis Grading

Seven days following CVB3-infection, the heart tissues were collected, sectioned and stained with hematoxylin and eosin (H&E). Sections were examined by two independent investigators in a blinded manner, and the severity of myocarditis was assessed by previously described 0–4 scale [27], in which 0 = no inflammation; 1 = one to five distinct mononuclear inflammatory foci with involvement of 5% or less of the cross-sectional area; 2 = more than five distinct mononuclear inflammatory foci, or involvement of over 5% but not over 20% of the cross-sectional area; 3 = diffuse mononuclear inflammation involving over 20% of the area, without necrosis; and 4 = diffuse inflammation with necrosis.

### Quantization of Viral Burden in Heart Tissues

Viral titers in the heart were determined as previously described [28]. Hearts were collected, weighed and homogenized in 400 µl 10%FBS-DMEM using a sterile glass mortar and pestle. After centrifugation at 14,000×g to remove cellular debris, 100 µl of the supernatant serially diluted in 10-fold increments, and incubated on confluent HeLa cell monolayer for 1 h at 37°C and 5% CO_2_ to allow virus attachment, and then incubated for 5 days to allow plaque formation. Viral titers were expressed as the mean lg TCID_50_/100 mg tissue±SEM.

### Cytokine Assays

Levels of TNF-α, IL-1β, IL-6, MCP-1 of cell culture supernatants and heart homogenates were determined by enzyme-linked immunosorbent assay (ELISA) (eBioscience) following the manufacturers’ instructions.

### Purification of Neonatal Murine Cardiac Myocytes

Cardiac myocytes from neonatal mice within 72 h of birth were prepared as previously reported [14]. Briefly, the hearts were minced finely and subjected to stepwise enzymatic digestion with 0.25% trypsin. The dissociated cells were washed with complete basal Eagle’s medium and depleted of endothelial cells and fibroblasts by two sequential 1 h adsorptions to plastic flasks at 37°C. The nonadherent myocytes were removed, washed once, resuspended in complete basal medium, and dispensed into tissue culture wells. After a period of 48 h, the myocytes were attached firmly to the plastic. According to observations on the shape and beating activity of the cells obtained, more than 95% cells were identified as cardiac myocytes. The cells were used as described below.

### Lentiviral Vector Construction, Production and Transduction

The A20 siRNA sequence was inserted into pLKO.1 (Addgene) to generate pLKO.1-shA20 that contained the shRNA targeting mouse A20 (5′-CAAAGCACUUAUUGACAGA-3′) [29]. The recombinant pseudotyped lentivirus was generated by co-transfection of three plasmids pLKO.1-shA20/pLKO.1-scramble shRNA, psPAX2 and pMD2.G (Addgene) into 293T cells using Lipofectamine Plus (Invitrogen) and concentrated by ultracentrifugation. The transduction of cardiac myocytes with lentiviral particle solution expressing either scramble shRNA (LV-ctrl) or A20 shRNA (LV-shA20) were described previously [30]. Cells (2.5×10^4^) were plated in 24-well plates. On the day of infection, the medium was removed and replaced with viral supernatant to which 5 µg/ml of polybrene had been added. 24 h after exposure, cells were washed with PBS twice and further incubated for 24 h in fresh culture medium.

### Western Blot and Kinase Assay

For western blot, 40 µg of extracted protein was fractionated by 8% to10% sodium dodecyl sulfate-polyacrylamide gels (SDS-PAGE). The blot was probed with 1 µg/ml primary Abs for p-p65, p65, IκBα, p-IκBα, (Cell Signaling Technology), β-actin, A20 (Santa Cruz Biotechnology). HRP-conjugated anti-rabbit or anti-mouse IgG (Southern Biotech) was used as a secondary Ab. Alternatively, cell lysates were immunoprecipitated with anti-IKKγ Ab (Cell Signaling Technology) by using protein A/G magnetic beads (Santa Cruz Biotechnology). The immunoprecipitates were incubated with GST-IκBα substrate at 37°C for 2 h. The phospho-GST-IκBα levels and IKKα/β protein expression shown as control were analyzed by immunoblotting as described [31], [32].The increased phospho-GST-IκBα expression indicating prolonged IKK kinase activity.

### Nuclear Factor-kappaB (NF-κB) DNA Binding Activity

Nuclear protein was isolated from cardiac tissues or cardiac myocytes using the Nuclear and Cytoplasmic Extraction Reagents Kit (NE-PER). NF-κB DNA binding activity in nuclei was determined using the NF-κB p65 transcription factor assay kit according to the manufacturer’s instructions (Cayman Chemical). Briefly, 10 µg nuclear extracts were added to the designated wells with complete transcription factor binding assay buffer (100 µl/well) and incubated overnight at 4°C. After 5 times washing with 200 µl wash buffer, 100 µl of 1∶100 diluted NF-κB p65 antibody was added for 1 h at room temperature. After washing, 100 µl of 1∶100 diluted goat anti-rabbit HRP conjugate was added to each well. 45 min later, 100 µl of transcription factor developing solution was added to each well and incubated for 15 min without light. Finally, 100 µl of stop solution was added and absorbance was measured at 450 nm.

### Assays of Endogenous Protein Ubiquitylation

Cardiac myocytes were pre-infected with adenovirus (Ad-A20, Ad-LacZ) or lentivirus (LV-shA20, LV-ctrl) for 48 h, then they were treated with CVB3 (multiplicity of infection, MOI = 10) for the indicated time, lysed in radio immunoprecipitation assay (RIPA) lysis buffer, boiled in 1%SDS, diluted 1∶10 in RIPA buffer, immunoprecipitated with antibodies specific for either RIP1 (Santa Cruz), RIP2 (Cell Signaling Technology) or TRAF6 (Santa Cruz), and analyzed by immunoblotting with antibodies to ubiquitin (Santa Cruz, P4D1), RIP1, RIP2 or TRAF6. The whole cell lysates (WCLs) were directly subjected to immunoblot analysis with specific antibodies as indicated.

### Statistical Analysis

Data were shown as the means±SEM. Statistical analysis of the data was performed using the GraphPad Prism (Version 5.0) statistical program. Means were compared using unpaired Student’s t-test. The survival rates of CVB3 infected mice were compared and analyzed with Kaplan-Meier plot. *P*<0.05 was considered statistically significant.

## Results

### Pro-inflammatory Cytokines were Markedly Up-regulated in CVB3 Infected Mice and Positively Correlated with the Severity of Acute Myocarditis

**Figure 1:**
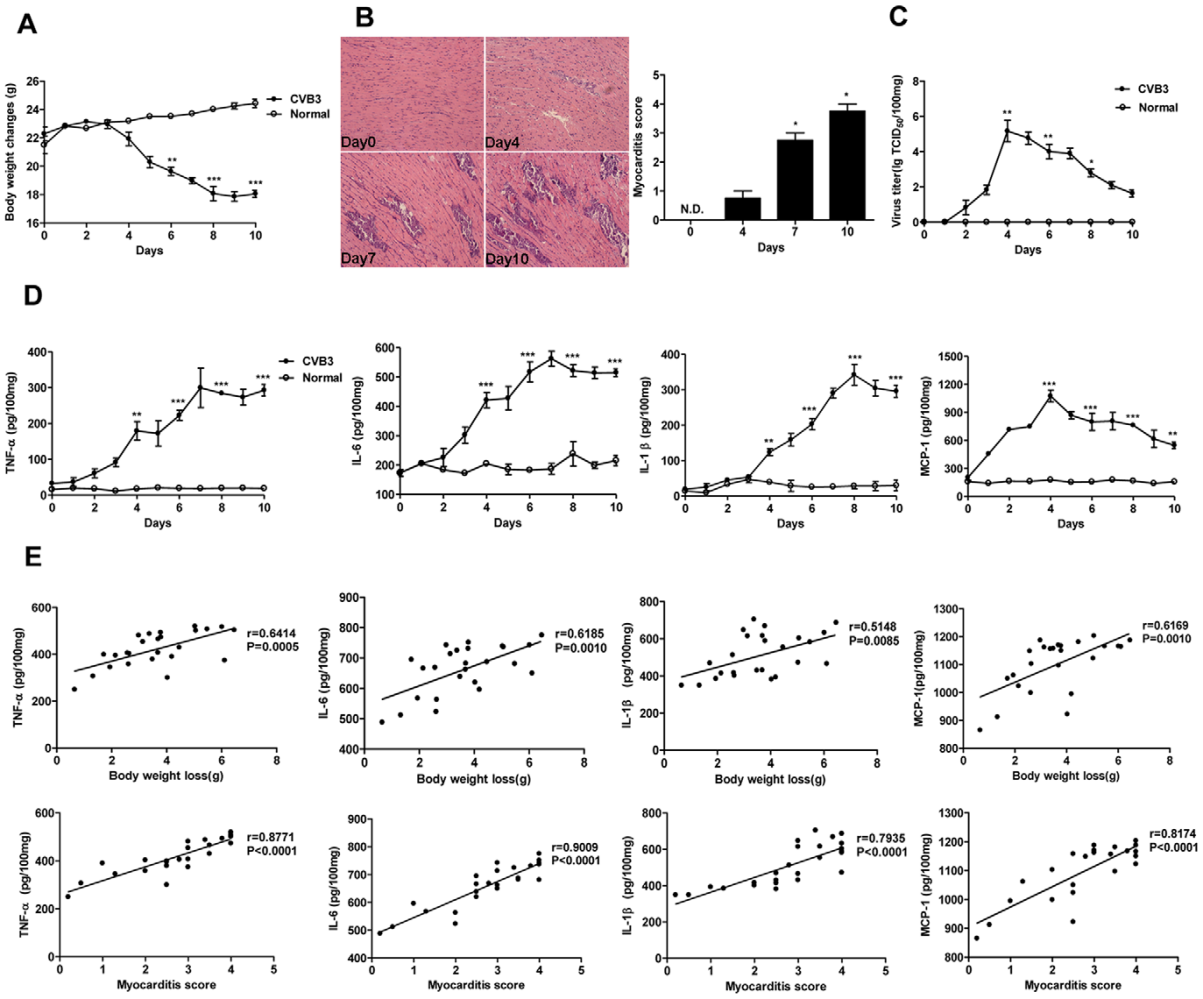
Expression kinetics of pro-inflammatory cytokines and their correlations with the severity of acute myocraditis. Male BALB/c mice were infected with 10^3^ TCID_50_ of CVB3 at day 0. (A) The body weight changes were monitored daily until day 10 post-infection. (B) Paraffin sections of heart tissues were prepared on day 0, 4, 7, 10 respectively and cardiac inflammation was revealed by H&E staining (magnification: ×200) (left). The severity of myocarditis was scored by a standard 0–4 scale according to the foci of mononuclear infiltration and myocardial necrosis (right). (C) Hearts were removed aseptically, weighed, and homogenized daily post-infection for TCID_50_ assay. (D) Hearts were collected and homogenized daily post-infection. The expression of pro-inflammatory cytokines (TNF-α, IL-6, IL-1β and MCP-1) were analyzed by ELISA assay. (E) Correlations between pro-inflammatory cytokines expression levels in cardiac tissues and measures of the severity of acute myocarditis (body weight loss or myocarditis pathological score) at day 7 following 10^3^ TCID_50_ CVB3 inoculation. Results were presented as the means±SEM of three separate experiments.*, *P*<0.05; **, *P*<0.01; ***, *P*<0.001. Each group contained 8 mice.

Male BALB/c mice were administered intraperitoneal injection with 10^3^ TCID_50_ CVB3 at day 0 to generate acute viral myocarditis model. The body weight changes were monitored daily until day 10 post-infection. And the histological analysis of heart sections was performed at day 0, 4, 7, 10 respectively. As shown in Figure 1A, the body weight of CVB3 infected mice were significantly and continuously decreased since day 4 (*P*<0.001) when compared with normal mice. Consistently, histopathology of cardiac tissues showed that the myocardial injury was apparently observed at day 4 and increasingly severe in the following days (*P*<0.05) (Figure 1B). Both the virus titer and pro-inflammatory cytokines expression including TNF-α, IL-6, IL-1β and MCP-1 in cardiac tissues were analyzed daily after CVB3 infection. The results showed that virus titer was peaked at day 4 (*P*<001) and then gradually reduced in the following days (Figure 1C). Differently, the expression of pro-inflammatory cytokines were robustly up-regulated at day 4 and persistently increasing from day 4 to day 10 in CVB3 infected mice (Figure 1D), suggesting that the pro-inflammatory cytokines might contribute to the pathogenesis of viral myocarditis.

To further investigate the involvement of pro-inflammatory cytokines in the pathological process of viral myocarditis, the correlations between the pro-inflammatory cytokines expression levels in cardiac tissues and two clinicopathological characteristics of viral myocarditis, including body weight loss and myocardial tissue pathological score, were respectively analyzed at day 7 following CVB3 infection. The results showed that the myocardial TNF-α, IL-6, IL-1β, MCP-1 levels were positively correlated with the body weight loss (TNF-α, r = 0.6414, *P* = 0.0005; IL-6, r = 0.6185, *P* = 0.001; IL-1β, r = 0.5148, *P* = 0.0085; MCP-1, r = 0.6169, *P* = 0.0010) and heart pathological score (TNF-α, r = 0.8771; IL-6, r = 0.9009; IL-1β, r = 0.7935; MCP-1, r = 0.8174; all *P*<0.0001) (Figure 1E), which confirming the essential participation of pro-inflammatory cytokines in the pathology of CVB3-induced myocarditis.

### Adenoviral-mediated Expression of A20 in Mice Decreased CVB3-induced Pro-inflammatory Cytokines Production

**Figure 2:**
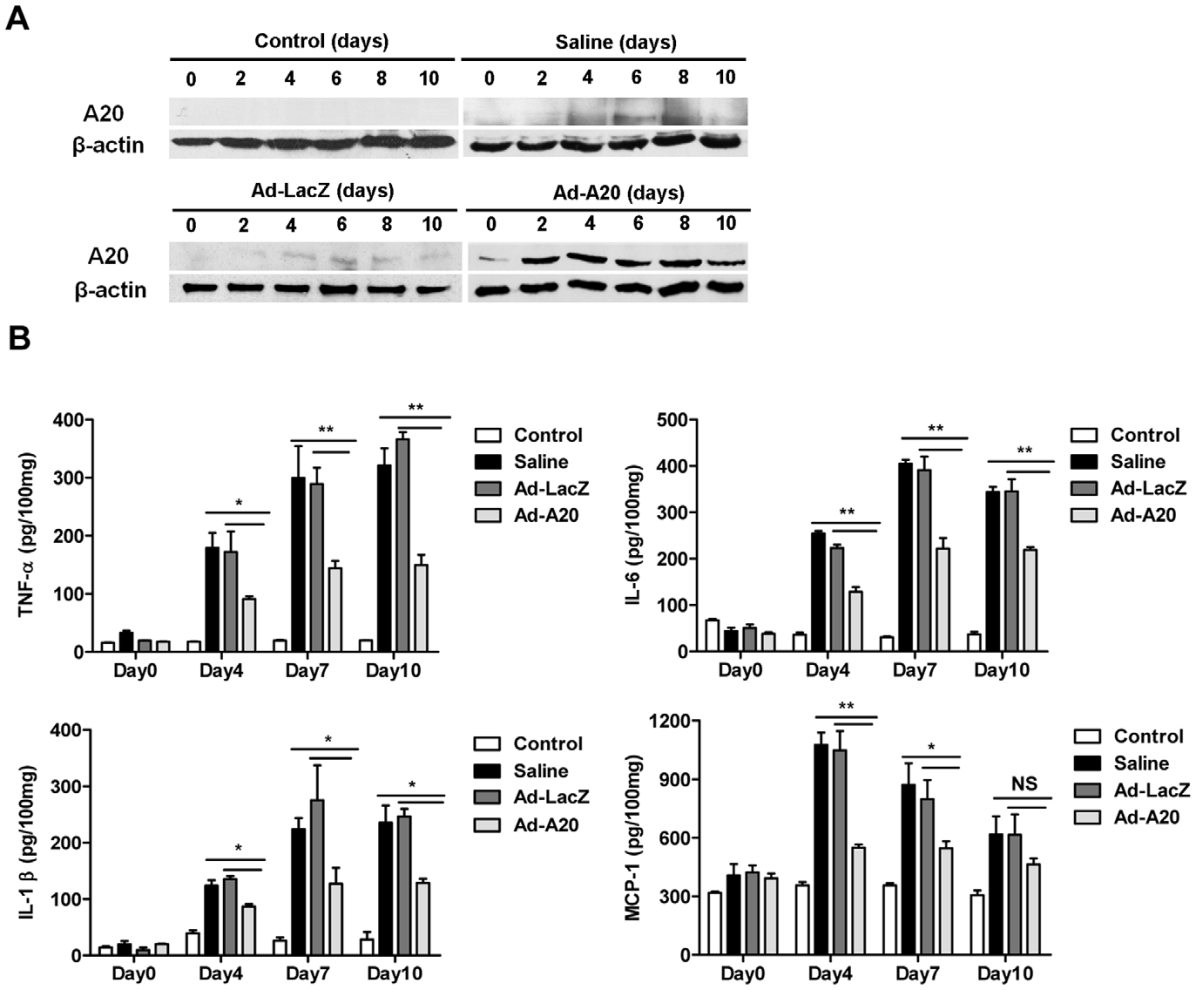
Suppression of pro-inflammatory cytokines production in CVB3 infected mice with Ad-A20 administration. Mice were intravenously injected with saline or 3×10^9^ pfu of either Ad-A20 or Ad-LacZ 2 days before 10^3^ TCID_50_ dose of CVB3 infection at day 0. Mice without infection were as control group. (A) Heart tissue homogenates prepared at indicated time points were subjected to western blot analysis with anti-A20 antibody. β-actin was used as loading control. Similar results were obtained in three independent experiments. (B) Heart tissue homogenates were prepared at the indicated time points. Protein levels of pro-inflammatory cytokines including TNF-α, IL-6, IL-1β and MCP-1 were determined by ELISA on day 0, 4, 7, 10 respectively post CVB3 infection. Data show the means±SEM of 6 mice per group. *, *P*<0.05; **, *P*<0.01; NS, no significance.

A20 has been reported as a negative regulator of inflammatory response [21], [22]. To determine whether A20 could inhibit pro-inflammatory cytokines production in CVB3-infected mice, a recombinant adenoviral vector expressing A20 (Ad-A20) was generated and Ad-LacZ was the control adenovirus. Groups of BALB/c mice were infected with 10^3^ TCID_50_ dose of CVB3 at day 0. To express A20 *in vivo*, mice were injected with 3×10^9 ^pfu of either Ad-A20 or control (Ad-LacZ) virus intravenously 2 days before CVB3 infection. Heart tissues were collected at two day intervals and A20 expression in heart homogenates was analyzed by western blot. The results showed that Ad-A20 administration resulted in high and sustained cardiac A20 protein expression from day 0 to 10, as compared with normal mice and CVB3 mice treated with saline or Ad-LacZ (Figure 2A).

Since A20 can be stable expressed in cardiac tissues of mice after Ad-A20 administration, the pro-inflammatory cytokines expression were measured by ELISA on day 0, 4, 7, 10 respectively following CVB3 infection. Compared with CVB3 mice treated with saline or Ad-LacZ, significant decreases of TNF-α (*P*<0.01), IL-6 (*P*<0.01) and IL-1β (*P*<0.05) were observed on both day 4, 7, 10 in the cardiac tissues of Ad-A20 injected mice and the expression levels of MCP-1 were significantly lower on day 4 (*P*<0.01) and 7(*P*<0.05) in the Ad-A20 treated CVB3 mice (Figure 2B). These results indicated that Ad-A20 treatment *in vivo* efficiently inhibited the production of pro-inflammatory cytokines in CVB3 infected mice.

### Treatment with Adenoviral Expressed A20 Alleviated CVB3-induced Myocarditis

**Figure 3:**
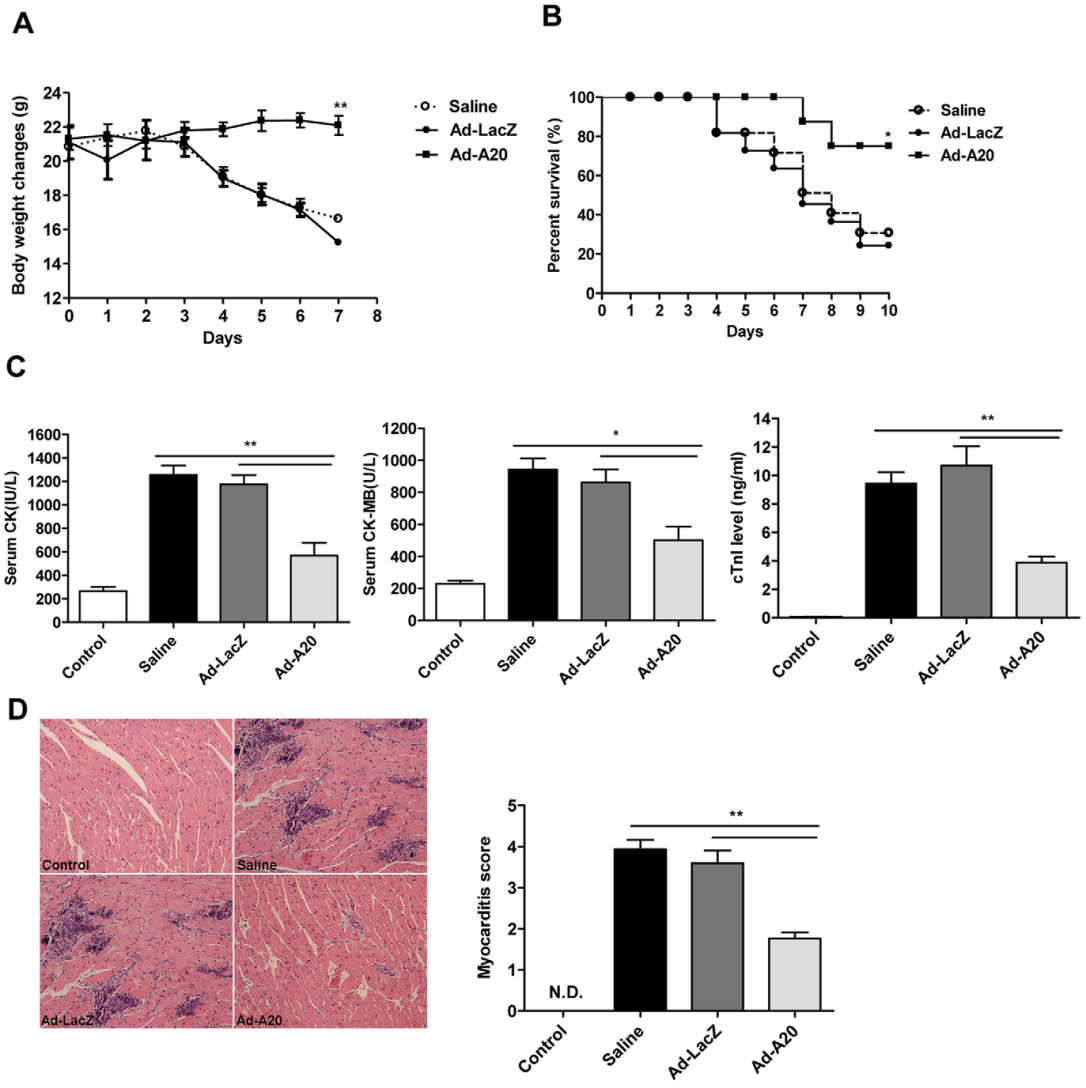
Ad-A20 administration mediated protection against CVB3-induced myocarditis. Mice were intravenously injected with saline or 3×10^9^ pfu of either Ad-A20 or Ad-LacZ 2 days before 10^3^ TCID_50_ dose of CVB3 infection. Mice without infection were as control group. (A and B) The body weight change (A) and survival rate (B) were respectively monitored daily until day 7 and day 10 post-infection. (C) Serological indices of myocarditis, the activity of CK, CK-MB and cTnI in mouse serum were detected on day 7 post-infection. (D**)** Paraffin sections of heart tissues were prepared on day 7 and cardiac inflammation was revealed by H&E staining (magnification:×200). The severity of myocarditis was scored by a standard 0–4 scale according to the foci of mononuclear infiltration and myocardial necrosis. Individual experiments were conducted 3 times with similar results, with 1 representative shown for each group. Data show the means±SEM of 6 mice per group. *, *P*<0.05; **, *P*<0.01; N.D., not detected.

To investigate the therapeutic effect of A20 on CVB3-induced acute myocarditis, mice were intravenously injected with saline or 3×10^9^ pfu of either Ad-A20 or Ad-LacZ virus 2 days before CVB3 inoculation. Parameters of the severity of myocarditis, including body weight loss, survival rate, serum creatine kinase (CK), CK-MB activity, cardiac troponin I (cTnI) level and pathological features of the sections of the heart tissues were carefully studied. As shown in Figure 3A and 3B, mice with saline or Ad-LacZ treatment underwent a dramatic and continuous loss of body weight as maximal to 32.5%, and more than 70% mice died within 10 days post-infection. On the contrary, mice receiving Ad-A20 had a little fluctuation in body weight (*P*<0.01) and a significantly improved survival rate (75%) (*P*<0.05). Consistently, serological indices of CK, CK-MB activities and cTnI levels were significantly decreased in mice with Ad-A20 treatment compared with Ad-LacZ or saline treated mice (Figure 3C), indicating a significantly reduced myocardial injury. Finally, histological analysis of heart sections revealed that CVB3 infected mice treated with saline or Ad-LacZ developed severe myocarditis on day 7 with diffuse inflammation, whereas Ad-A20 treatment led to a significant remission of myocarditis showing few restricted mononuclear inflammation foci and tiny necrosis (*P*<0.05) (Figure 3D). All the above data indicated that Ad-A20 treatment could effectively protect mice from lethal myocarditis caused by CVB3 infection.

### A20 Inhibited NF-κB Signaling Activation to Suppress CVB3-induced Pro-inflammatory Cytokines Production

**Figure 4:**
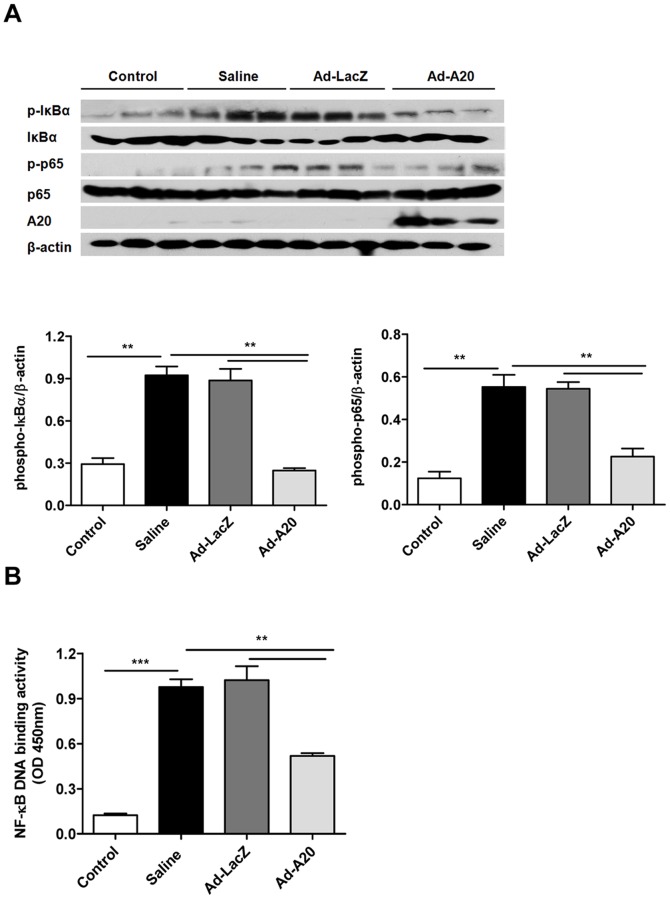
The inhibitory effect of A20 on CVB3-induced NF-κB activation in mice. Mice were intravenously injected with saline or 3×10^9^ pfu of either Ad-A20 or Ad-LacZ 2 days before 10^3^ TCID_50_ dose of CVB3 inoculation. Mice without infection were as control group. Heart homogenates were prepared on day 4 after CVB3 infection. (A) The phosphorylation of p65 and IκBα were assessed by western blotting. The data were collected from three mice for each group. Above, representative western blots; Below, quantitative results, the band intensity was measured and the ratio of phospho-IκBα and phospho-p65 to β-actin was calculated. Data were expressed as means±SEM. (B) NF-κB DNA binding activity was analyzed by NF-κB p65 transcription factor assay kit. Data show the means±SEM of 6 mice per group. **, *P*<0.01; ***, *P*<0.001.

Virus infection leads to the activation of natural immune signaling pathways. On the basis of the knowledge that nuclear factor-kappaB (NF-κB) signaling induces transcription of various pro-inflammatory mediators [33], we hypothesized that A20 would inhibit NF-κB activation induced by CVB3 *in vivo* to restrict the inflammatory response. Mice were intravenously injected with saline or 3×10^9^ pfu of either Ad-A20 or Ad-LacZ virus 2 days before CVB3 inoculation. The cytoplasmic and nuclear protein was extracted from heart homogenates at day 4. There was a significant increase in the levels of the phosphorylation of IκBα and p65-NF-κB subunit from heart tissues of CVB3-infected mice treated with saline, which are indicators of NF-κB signaling activation (Figure 4A), as well as an increase in the binding activity of heart nuclear extracts to a NF-κB consensus sequence compared with control mice (Figure 4B) (*P*<0.001). However, heart tissues from CVB3-infected mice treated with Ad-A20 showed lower levels of the phosphorylation of IκBα and p65 when compared with saline or Ad-LacZ treated group mice. The NF-κB DNA binding activity was also significantly decreased after Ad-A20 treatment (*P*<0.01). These results indicated that A20 may interfere NF-κB signaling pathway for anti-inflammation since the early stage in CVB3 induced myocarditis model.

**Figure 5:**
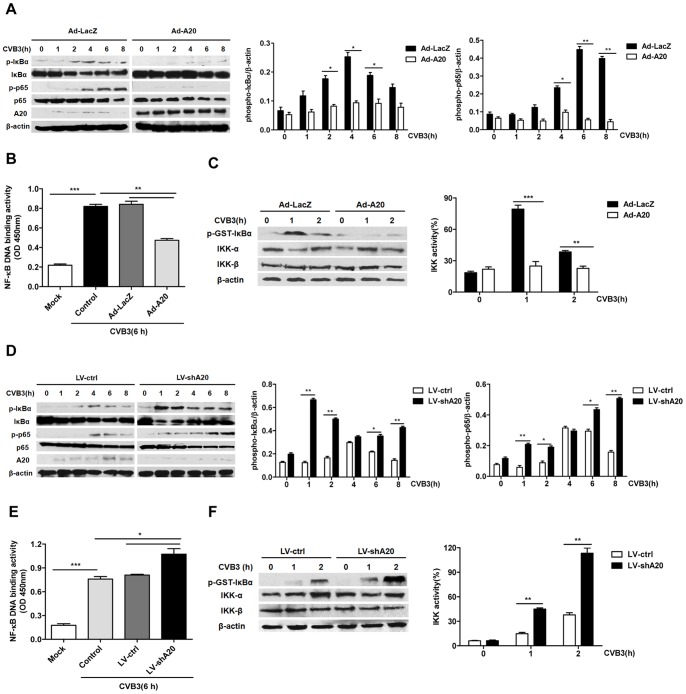
The effect of A20 on CVB3-induced NF-κB signaling in cardiac myocytes. Cardiac myocytes were pre-infected with adenovirus (Ad-LacZ or Ad-A20) to over-express A20 or lentivirus (LV-ctrl or LV-shA20) to knock down endogenous A20 for 48 h, then exposed to CVB3 (MOI = 10) for the indicated time. (A and D) Cell lysates were examined for phosphorylation of IκB-α, p65 and protein expression of total IκB-α, p65 and A20. β-actin was probed as the loading control. Left, representative western blots; Right, quantitative results, the band intensity was measured and the ratio of phospho-IκBα and phospho-p65 to β-actin was calculated. (B and E) NF-κB p65 DNA binding activity of nuclear extracts from cardiac myocytes was measured using the NF-κB p65 transcription factor assay kit. (C and F) Cell lysates were harvested at the indicated time, immunoprecipitated with anti-IKKγ antibody, incubated with GST-IκBα substrate, and analyzed by immunoblotting for phospho-GST-IκBα levels. Left, representative western blots; Right, quantitative results. Each assay was done 3 times. Values were presented as the means±SEM. *, *P*<0.05; **, *P*<0.01; ***, *P*<0.001.

In the early stage of viral myocarditis, CVB3 infect host cardiomyocytes and trigger innate immune signaling, which is required for the induction of subsequent cardiac inflammatory response and cardiomyopathy [34]. To investigate whether A20 could restrict CVB3-induced NF-κB signaling activity *in vitro*, the primary cardiac myocytes were purified from neonatal BALB/c mice and pre-infected with adenovirus Ad-A20 or Ad-LacZ for 48 h. Then the cardiac myocytes were treated with CVB3 for 0, 1, 2, 4, 6, 8 h. Cell lyses and nuclear extracts were prepared at the indicated time points and subjected to western blot analysis and ELISA-based transcription factor assay. It was found that CVB3 triggered the phosphorylation of IκBα and p65-NF-κB subunit. A20 over-expression mediated by Ad-A20 inhibited phosphorylation of cytosolic IκBα and p65 (Figure 5A). The result of NF-κB DNA binding activity also showed that CVB3 significantly increased NF-κB p65 DNA binding activity (*P*<0.001), A20 over-expression significantly attenuated this effect (*P*<0.01) (Figure 5B). Because phosphorylation of IκBα is mediated through IκBα kinase (IKK) activation, then the activity of IKK signalosome was analyzed by performing kinase assays on lysates from CVB3 stimulated cardiac myocytes, which were pre-infected with Ad-A20 or Ad-LacZ. The results showed that Ad-A20 infected cells displayed impaired IKK activity when compared to Ad-LacZ infected cells (*P*<0.01) (Figure 5C). These data indicated that A20 over-expression inhibited CVB3-induced NF-κB signaling activation.

To further confirm that A20 was physiologically required for restricting CVB3-induced NF-κB signaling, the cardiac myocytes were infected with lentivirus expressed shRNA specifically knock down endogenous A20 (LV-shA20) or its control (LV-ctrl), then the cells were treated with CVB3 for the indicated time. The phosphorylation of IκBα and p65, NF-κB DNA binding activity and the IKK activity were analyzed. It was found that the phosphorylation of IκBα and p65 induced by CVB3 were elevated after A20 was knock down (Figure 5D). Both the NF-κB DNA binding activity (*P*<0.05) (Figure 5E) and IKK activity (*P*<0.01) (Figure 5F) were exaggerated when compared with the control cells. These findings suggested that A20 was physiologically required for restricting CVB3-induced NF-κB signaling.

**Figure 6:**
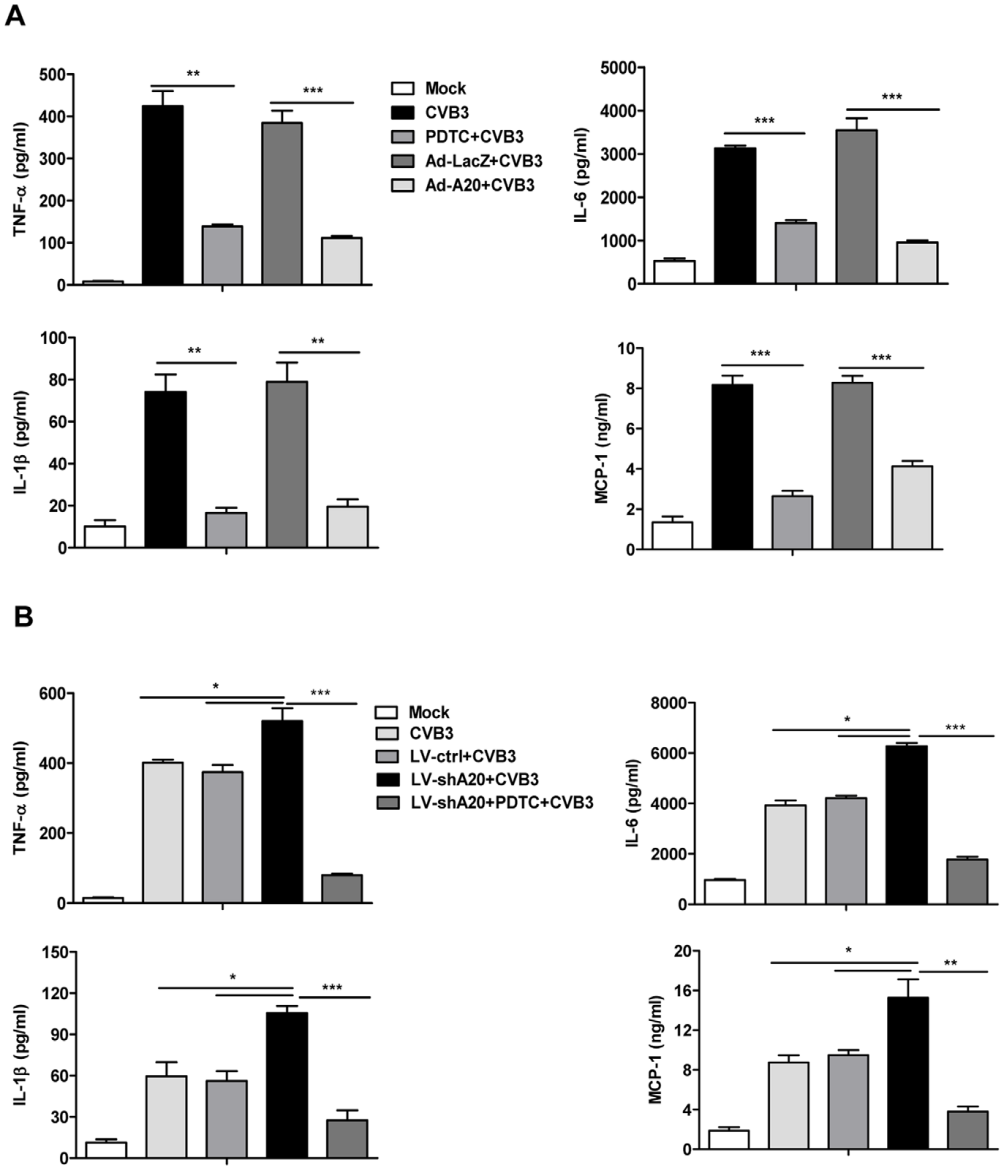
The effect of A20 on CVB3-induced pro-inflammatory cytokines production in cardiac myocytes. (A) Cardiac myocytes were pre-infected with adenovirus (Ad-LacZ or Ad-A20) for 48 h, or pretreated with a NF-κB inhibitor PDTC (10 µmol/L) for 1 h. Then they were exposed to CVB3 (MOI = 10) for 24 h. The culture medium was collected and cytokines expression was assayed by ELISA. (B) Cardiac myocytes were pre-infected with lentivirus (LV-ctrl or LV-shA20) for 48 h, or plus treated with a NF-κB inhibitor PDTC (10 µmol/L) for 1 h. Then they were exposed to CVB3 (MOI = 10) for 24 h. The culture medium was collected and cytokines expression was assayed by ELISA. Data were presented as the means±SEM of three separate experiments. *, *P*<0.05; **, *P*<0.01; ***, *P*<0.001.

To examine the effect of A20 on the production of pro-inflammatory cytokines in CVB3-infected cardiac myocytes, cells were prior infected with Ad-A20 or its control Ad-LacZ for 48 h, or pretreated with a NF-κB inhibitor PDTC (10 µmol/L) for 1 h. Then they were treated with CVB3 for 24 h. The culture medium was collected and cytokines expression was assayed by ELISA. As shown in Figure 6A, CVB3 infection resulted in robust expression of TNF-α, IL-6, IL-1β and MCP-1 in cardiac myocytes, which were significantly decreased when the NF-κB signaling was inhibited by PDTC. Likewise, A20 over-expression mediated by Ad-A20 diminished the expression of TNF-α, IL-6, IL-1β and MCP-1 by 75.3±0.1% (*P*<0.001), 74.8±0.8% (*P*<0.001), 55.1±0.3% (*P*<0.01), 72.7±0.5% (*P*<0.001) respectively. On the contrary, their expression levels were elevated when A20 was knock down by LV-shA20. But this elevation was markedly impaired by PDTC treatment (Figure 6B). In addition, no significant effect of A20 on the cell viability of cardiac myocytes in the culture medium was observed (data not shown).

Collectively, all these data indicated that A20 suppressed CVB3-induced inflammatory cytokines production via inhibiting NF-κB signaling.

### Reduced Endogenous TRAF6 Ubiquitylation Conferred the Inhibitory Effect of A20 on CVB3-induced NF-κB Signaling

**Figure 7:**
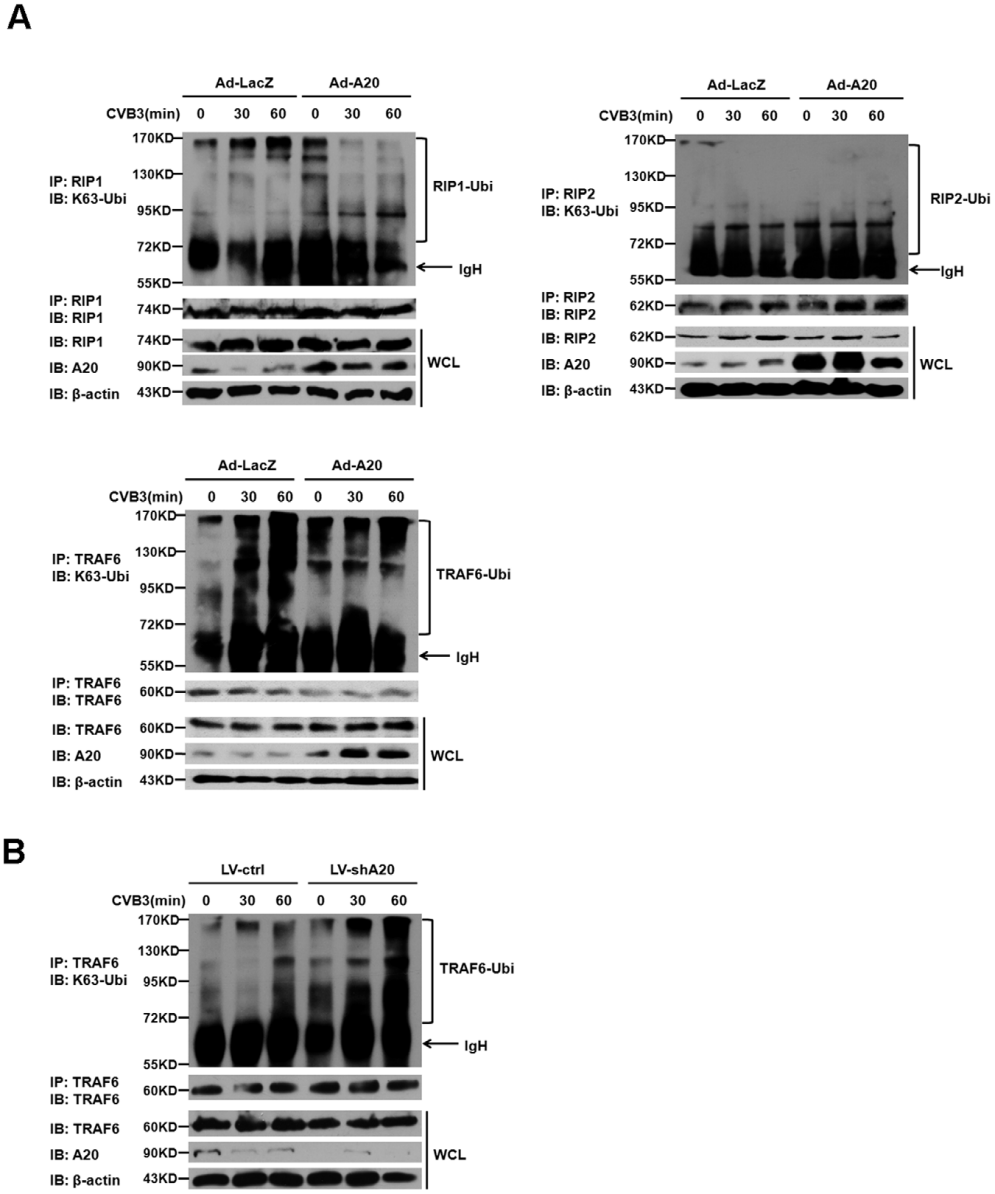
The effect of A20 on endogenous TRAF6 ubiquitylation in CVB3-infected cadiac myocytes. (A) Cardiac myocytes were pre-infected with adenovirus Ad-LacZ or Ad-A20 for 48 h, then they were treated with CVB3 (MOI = 10) for the indicated time, lysed in RIPA buffer, immunoprecipitated with RIP1, RIP2 and TRAF6 antibody. Immunoprecipitated protein complex was then analyzed by immunoblotting for ubiquitin. Immunoblotting for RIP1, RIP2 and TRAF6 on immunoprecipitates was shown below as a control. The whole cell lysates (WCLs) were subjected to immunoblot analysis with specific antibodies as indicated. (B) Cardiac myocytes were pre-infected with lentivirus LV-ctrl or LV-shA20 for 48 h, then they were treated with CVB3 (MOI = 10), lysed in RIPA buffer, immunoprecipitated with TRAF6 antibody as described above. Data are representative of three independent experiments.

To better understand the molecular mechanism by which A20 inhibited CVB3-induced NF-κB signaling, we considered that A20 is an ubiquitin-editing enzyme that has been reported to directly remove K63-linked polyubiquitin chains of receptor interacting protein 1 (RIP1), TNF receptor associated factor 6 (TRAF6) and RIP2, which are critical in signaling to the IKK complex activation and downstream phosphorylation of IκBα [35]–[37]. The above data showed that A20 was able to restrict IKK activity and the phosphorylation of IκBα induced by CVB3. To determine which targeting protein for A20 may involve in its inhibitory effect on CVB3 activated NF-κB signaling, cardiac myocytes pre-infected with Ad-LacZ or Ad-A20 were treated with CVB3, lysed at the indicated time point, immunoprecipitated with specific antibodies for RIP1, TRAF6 and RIP2 respectively, and the K63-linked ubiquitination status of these proteins were tested by immunoblotting for ubiquitin. The whole cell lysates (WCLs) were directly subjected to immunoblot analysis of RIP1, TRAF6, RIP2, A20 and β-actin as loading control. These experiments revealed that endogenous TRAF6, rather than RIP1 or RIP2 was obviously ubiquitylated in CVB3 infected cardiac myocytes. Strikingly, the ubiquitylated levels of TRAF6 were reduced in A20 over-expressed cardiac myocytes (Figure 7A), suggesting that A20 may deubiquitylate TRAF6 to inhibit CVB3 activated NF-κB signaling. To further examine whether A20 may physiologically regulate TRAF6 ubiquitylation in CVB3-induced NF-κB signaling, the same endogenous TRAF6 ubiquitylation assay was performed in A20 knock down cardiac myocytes with lentivirus (LV-shA20) infection. The results showed that A20 knock down led to an increase of CVB3-induced ubiquitylation of TRAF6 (Figure 7B). All these data indicated that A20 restricted endogenous TRAF6 ubiquitylation, by which it suppressed CVB3-induced NF-κB signaling.

## Discussion

Viral myocarditis is characterized by excessive inflammation of myocardium leading to heart injury following enterovirus infections. Local secretion of cytokines and chemokines by cardiomyocytes and infiltrated inflammatory cells over the course of virus infection is important in determining the pathogenesis of viral myocarditis. During the first 1–4 day after virus infection, virus are replicated in cardiac myocytes and trigger innate immune signaling, which contribute to the increasing expression of pro-inflammatory cytokines including TNF-α, IL-6, IL-1β and chemokines. These cytokines are crucial for the recruitment and activation of immune cells. At later stage of infection, from day 5 to approximately day 14, immune cells from the adaptive immune system accumulate in the infected heart and strongly augment the expression of pro-inflammatory cytokines, result in the massive inflammation and aggravated injury in heart [34], [38]. In our study, histopathology of cardiac tissues revealed by H&E showed that the myocardial inflammation was increasingly severe since day 4 in CVB3-infected mice, accompanying by continuously bodyweight loss. However, the virus titer in the cardiac tissues was peaked at day 4 and then gradually reduced in the following days. Differently, the expression of the pro-inflammatory cytokines were robustly up-regulated at day 4 and persistently increasing in the following days in CVB3 infected mice, and were positively correlated with the severity of CVB3-induced viral myocarditis, further confirming their pathologic role in the progress of viral myocarditis. Our data were consistent with previous studies and indicated that modulation of pro-inflammatory cytokines production since the early stage could be effective for the treatment of viral myocarditis.

Here, our study found that intravenous injection *in vivo* with adenovirus expressed A20 (Ad-A20) 2 days before CVB3 inoculation could significantly decrease the expression of pro-inflammatory cytokines in cardiac tissues on day 4, 7, and 10 and protect mice against viral myocarditis, as demonstrated by invariant body weight, improved survival rate, less increased serological CK, CK-MB, cTnI levels and less myocardical inflammation. Our findings may provide a new therapeutic strategy for the treatment of viral myocarditis.

A20 is a cytoplasmic protein that was originally identified as a TNF-inducible protein in endothelial cells and has been characterized as a central regulator of immunopathology [21]. Genetic studies show that A20 plays important roles in human autoimmune diseases. Polymorphisms in or near the human tnfaip3 (A20) gene are associated with rheumatoid arthritis, Crohn’s disease and systemic lupus erythematosus (SLE) [39]–[41]. Increasing experimental studies demonstrate the favorable effects of A20 against inflammatory response. Over-expression of A20 is protective against atherosclerosis in mice [26]. Prior injection of adenovirus expressed A20 has shown a potent therapeutic effect in an allergic airway inflammation model and a collagen-induced arthritis model [24], [25]. A20 also protects pancreatic islets from cytokine toxicity [42], and the heart from myocardial infarction [31]. A recent report showed that specific deletion of A20 in myeloid cells protected mice against lethal influenza A virus infection [43]. Here our study provided the evidence that A20 over-expression could have protective effect on viral myocarditis.

Virus infection leads to the activation of natural immune signaling pathways. NF-κB pathway has been considered a prototypical pro-inflammatory signaling pathway, based on the activation of large pro-inflammatory genes including cytokines, chemokines, and adhesion molecules [33], which contribute to the pathogenesis of viral myocarditis. A NF-κB inhibitor, SUN C8079 has ever been used *in vivo* to prevent the development of myocarditis caused by the encephalomyocarditis virus (EMCV) and inhibit the expression of pro-inflammatory cytokines in cardiac tissues [44]. Our results showed that NF-κB signaling was significantly inhibited in CVB3-infected mice received Ad-A20 treatment, evidenced by the reduced phosphorylated levels of IκBα and p65 and the impaired NF-κB DNA binding activity, and the experiments in cardiac myocytes of A20 over-expression or knock down demonstrated that A20 was physiologically required to inhibit CVB3-induced NF-κB signaling, which resulted in lower expression levels of pro-inflammatory cytokines. Our study indicated that innate immune signaling pathways could be novel targets for the treatment of viral myocarditis. Interestingly, we observed that myocardial virus titer was reduced in Ad-A20 treated mice on day 4, but had no significant change on day 7 or day 10 (Figure S1), suggesting the inhibition of NF-κB signaling may be beneficial to restrict virus replication at early stage of viral myocarditis, consistent with previous reports which showed that NF-κB inhibitor BAY11-7085 treatment in CVB3 infected HL-1 cardiomyocytes and HeLa cells could reduce viral progeny release [45], [46]. Further investigations are needed to clarify the molecular mechanisms utilized by CVB3 to interfere with NF-κB pathway, which may enable us to exploit NF-κB as a new weapon against viral myocarditis.

Previous studies have elucidated that A20 plays an essential role in the inhibition of NF-κB signaling triggered by TNF-TNF receptor (TNFR), IL-1-IL-1R, lipopolysaccharide (LPS)-toll like receptor 4 (TLR4) and muramyl dipeptide (MDP)-nucleotide-binding oligomerization domain containing 2 (NOD2) [47]. A20 has also been reported to restrict influenza virus induced NF-κB signaling in bronchial epithelial cells [48]. Here our findings showed that A20 was physiologically required to inhibit CVB3-induced NF-κB signaling, implying that A20 could also regulate innate immune signaling in response to virus infection.

The molecular mechanism responsible for the NF-κB inhibitory function of A20 has been clarified by the elucidation of A20 as an ubiquitin-editing protein with dual functions: a deubiquitinating enzyme (DUB) activity mediated by its N-terminal ovarian tumor (OTU) domain and E3 ubiquitin ligase activity mediated by the C-terminal zinc finger containing domain [21]. In the case of TNF signaling, A20 terminates NF-κB activation with its DUB activity that removes K63-linked polyubiquitin chains from RIP1 and its E3 ubiquitin ligase activity that promotes RIP1 K48-linked polyubiquitination, which triggers the proteasome-mediated degradation of RIP1 [35]. A20 can also dismantle K63-linked polyubiquitin chains from TRAF6 and RIP2, similarly turning off NF-κB activation induced by TLR4 and NOD2 respectively [36], [37]. Here we found that endogenous TRAF6, rather than RIP1 or RIP2 was ubiquitylated in CVB3-infected cardiac myocytes. And the ubiquitylated levels of TRAF6 were reduced in A20 over-expressed cardiac myocytes, but elevated in A20 knock down cardiac myocytes, suggesting that A20 may deubiquitylate TRAF6 to inhibit CVB3 activated NF-κB signaling. To our knowledge, this is the first study to demonstrate that A20 is required to inhibit CVB3 activated NF-κB signaling by restricting endogenous TRAF6 ubiquitylation. We do not exclude there may other targeting molecules for A20 involve in its inhibitory effect on CVB3 activated NF-κB signaling and further investigations will be needed.

The present study demonstrated that intravenous injection with adenovirus expressed A20 (Ad-A20) into mice 2 days before CVB3 inoculation effectively alleviated the severity of viral myocarditis, which was through down regulation of CVB3 induced NF-κB signaling and consequent reduction of pro-inflammatory cytokines production. Our results suggested that A20 could interfere innate signaling pathway in CVB3 infected cardiomyocytes since the early stage and attenuate subsequent excessive inflammatory response in the heart. However, we do not exclude the possible effect of A20 on the biological behavior of cardiomyocytes and the immune cells, which might also account for the protective effect of A20 on myocarditis. In fact, studies existed have shown that A20 over-expression could prevent cardiomyocytes apoptosis, cardiac hypertrophy and fibrosis [31], [49]. Here, we also observed the effect of A20 on the immune cells in this myocarditis model by performing immunohistology with cardiac sections and flow cytometric analysis with single-cell suspensions of cardiac cells on day 7. The results showed that Ad-A20 treatment could effectively reduce CD45, CD3, CD11b positive inflammatory cells infiltrating into heart (Figure S2) and inhibit NF-κB signaling in the immune cells isolated from spleens of CVB3 infected mice (Figure S3). Our data suggested that A20 had anti-inflammatory effect via inhibiting NF-κB signaling on both cardiomyocytes and immune cells in CVB3 infected mice. Of course, the precise molecular mechanisms by which A20 prevents mice from CVB3-induced myocarditis and the cell types that A20 mainly modulates in *vivo* undoubtedly deserve successive studies.

In conclusion, we report for the first time that delivery of adenovirus expressed A20 *in vivo* could abrogate CVB3-induced cardiac inflammation and alleviate the severity of myocarditis. The suppression of CVB3-induced inflammatory response by A20 was due to the inhibiting of NF-κB signaling pathway. A20 was physiologically required to inhibit CVB3-induced NF-κB signaling through restricting endogenous TRAF6 ubiquitylation. Our findings may provide an insight into better understanding of the underlying immune-pathological mechanism in CVB3-induced myocarditis, and constitute the first preclinical data indicating that A20 can control CVB3-induced myocarditis. This strategy may be a clinically relevant and feasible therapeutic strategy for patients suffering from CVB3-induced myocarditis or other inflammatory heart diseases.

## Supporting Information

Figure S1 **Titration of the myocardial virus in CVB3 infected mice after Ad-A20 administration.** Mice were intravenously injected with saline or 3×10^9^ pfu of either Ad-A20 or Ad-LacZ 2 days before 10^3^ TCID_50_ dose of CVB3 infection at day 0. Hearts were removed aseptically, weighed, and homogenized on day 0, 4, 7 and 10 post-infection for TCID_50_ assay. Data show the means±SEM of 6 mice per group. **, *P*<0.01; N.D., not detected; NS, no significance.
(TIF)

Figure S2 **Attenuation of inflammatory cells infiltration in the heart of CVB3 mice with Ad-A20 administration.** Mice were intravenously injected with saline or 3×10^9^ pfu of either Ad-A20 or Ad-LacZ 2 days before 10^3^ TCID_50_ dose of CVB3 infection at day 0. Mice without infection were as control group. (A) Hearts were collected on day 7 post-infection. Cardiac sections were stained with anti-CD3 antibody to identify T lymphocytes and anti-CD11b antibody to identify monocytes. Micrographs show immunostaining results from a representative animal per group. Each group contained 5 mice. (B) single-cell suspensions of cardiac cells were prepared by digesting small pieces of heart at day 7 post-infection. The cells were collected and stained for immune cells marker, including CD45, CD3 and CD11b. Then the stained cells were subjected to flow cytometric analysis. Isotype Ab staining has been subtracted from each set of data in the graphs. We used the percentage of total cardiac cells to allow comparison of the inflammatory cells present in cardiac infiltrates between different groups. Flow cytometry was performed on cardiac cells from all animals in each group (n = 5). The histograms were representative data stained for each marker. (C) Quantitative results showed the percentage of CD45, CD3, CD11b positive cells in the heart of CVB3 mice (n = 5). **, *P*<0.01.
(TIF)

Figure S3 **The inhibitory effect of A20 on the production of inflammatory cytokines from CVB3 infected immune cells. (A)** Mouse splenocytes isolated from spleens were pre-infected with adenovirus (Ad-LacZ or Ad-A20) to over-express A20 or not. Then they were exposed to CVB3 (MOI = 10) for 24 h. The culture medium was collected and cytokines expression was assayed by ELISA. Data were presented as the means±SEM of three separate experiments. *, *P*<0.05; **, *P*<0.01; ***, *P*<0.001. (B) Mice were intravenously injected with saline or 3×10^9^ pfu of either Ad-A20 or Ad-LacZ 2 days before 10^3^ TCID_50_ dose of CVB3 infection at day 0. Mice without infection were as control group. 7 days post-infection, splenocytes were isolated from spleens and lysed with RIPA for western blot analysis with the indicated antibodies. NF-κB DNA binding activity was analyzed by NF-κB p65 transcription factor assay kit. Data show the means±SEM of 5 mice per group. **, *P*<0.01.
(TIF)
