# Supplementary figures and images for: A20 (TNFAIP3) Alleviates CVB3-Induced Myocarditis via Inhibiting NF-κB Signaling
Source: PLoS One. 2012 Sep 28;7(9):e46515. doi: 10.1371/journal.pone.0046515 (PMC3460927; doi:10.1371/journal.pone.0046515)

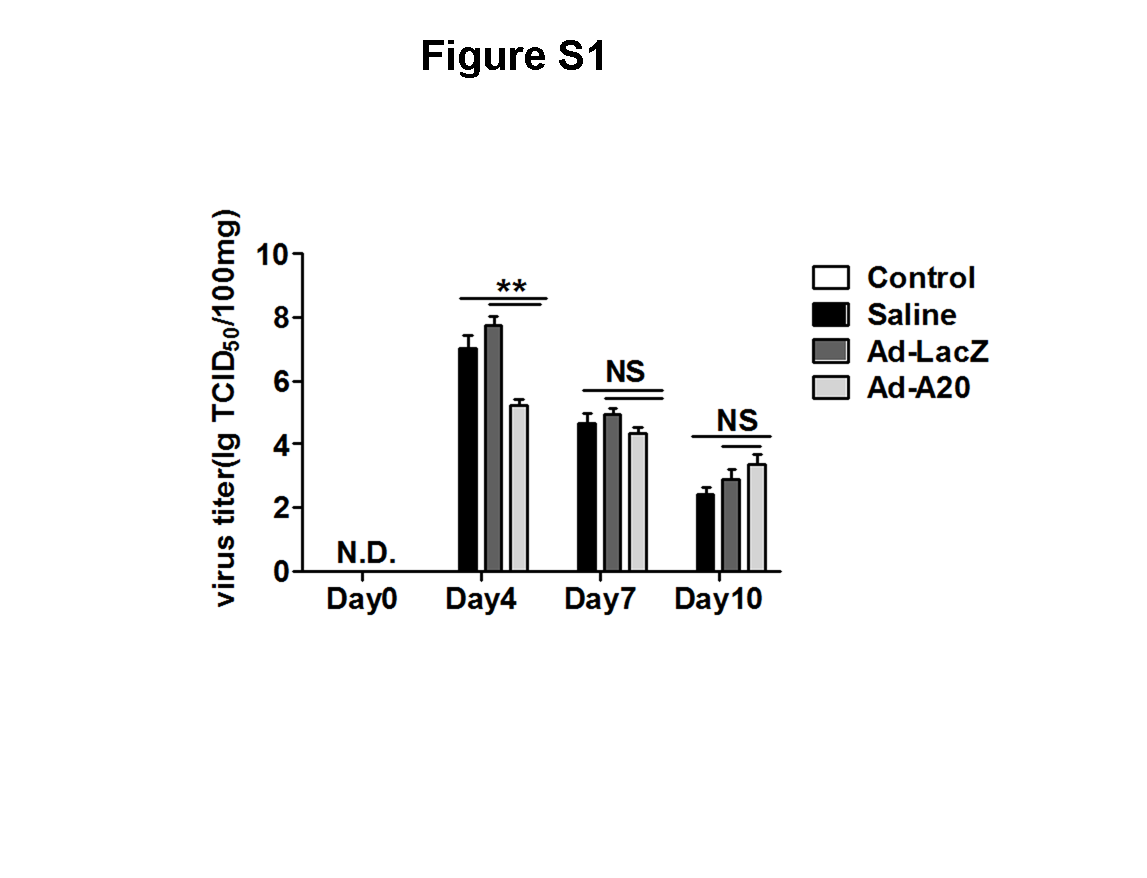

Supplement: Figure S1 — Titration of the myocardial virus in CVB3 infected mice after Ad-A20 administration. Mice were intravenously injected with saline or 3×10^9^ pfu of either Ad-A20 or Ad-LacZ 2 days before 103 TCID50 dose of CVB3 infection at day 0. Hearts were removed aseptically, weighed, and homogenized on day 0, 4, 7 and 10 post-infection for TCID50 assay. Data show the means±SEM of 6 mice per group. **, P<0.01; N.D., not detected; NS, no significance. (TIF) [file pone.0046515.s001.tif]

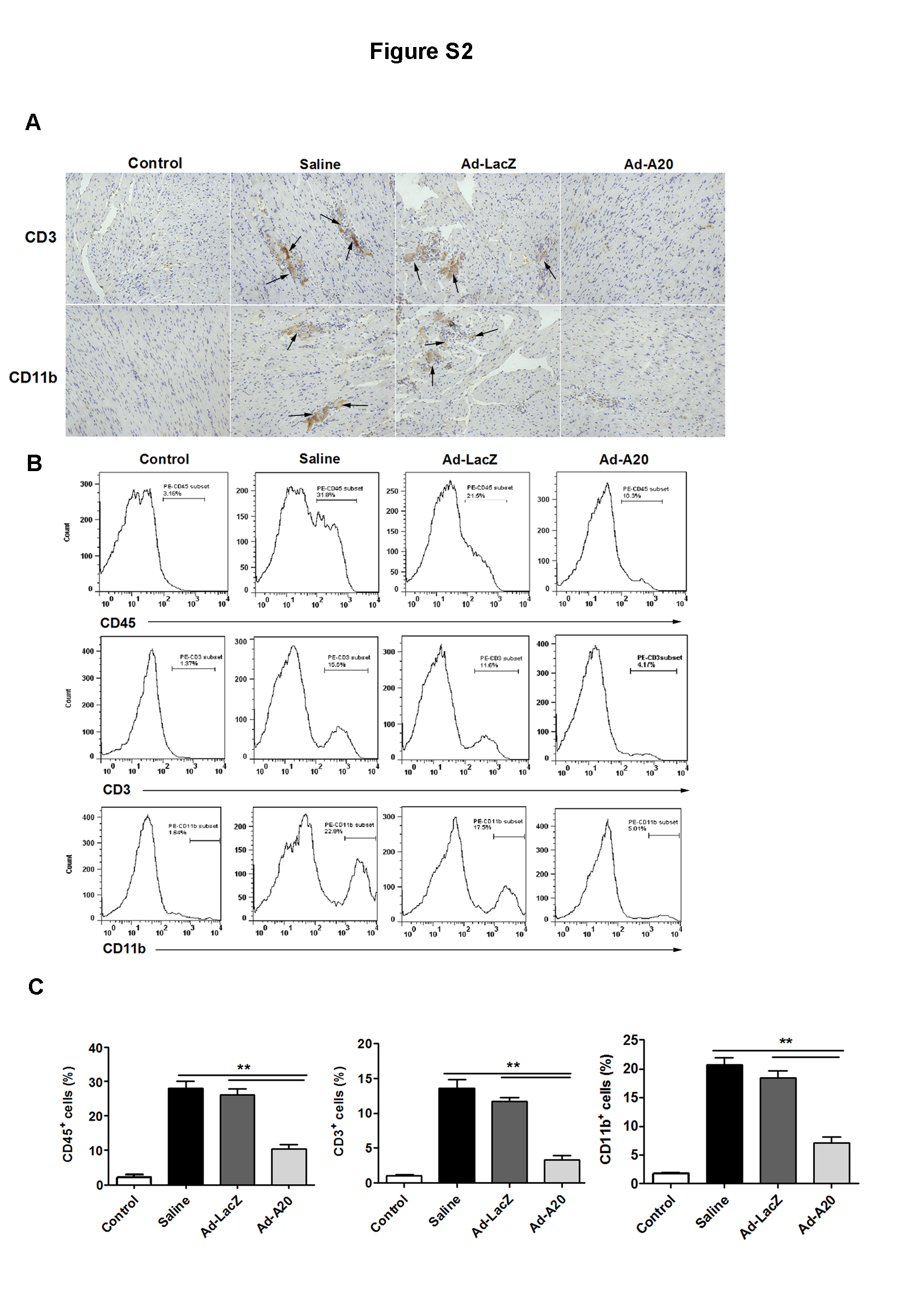

Supplement: Figure S2 — Attenuation of inflammatory cells infiltration in the heart of CVB3 mice with Ad-A20 administration. Mice were intravenously injected with saline or 3×10^9^ pfu of either Ad-A20 or Ad-LacZ 2 days before 103 TCID50 dose of CVB3 infection at day 0. Mice without infection were as control group. (A) Hearts were collected on day 7 post-infection. Cardiac sections were stained with anti-CD3 antibody to identify T lymphocytes and anti-CD11b antibody to identify monocytes. Micrographs show immunostaining results from a representative animal per group. Each group contained 5 mice. (B) single-cell suspensions of cardiac cells were prepared by digesting small pieces of heart at day 7 post-infection. The cells were collected and stained for immune cells marker, including CD45, CD3 and CD11b. Then the stained cells were subjected to flow cytometric analysis. Isotype Ab staining has been subtracted from each set of data in the graphs. We used the percentage of total cardiac cells to allow comparison of the inflammatory cells present in cardiac infiltrates between different groups. Flow cytometry was performed on cardiac cells from all animals in each group (n = 5). The histograms were representative data stained for each marker. (C) Quantitative results showed the percentage of CD45, CD3, CD11b positive cells in the heart of CVB3 mice (n = 5). **, P<0.01. (TIF) [file pone.0046515.s002.tif]

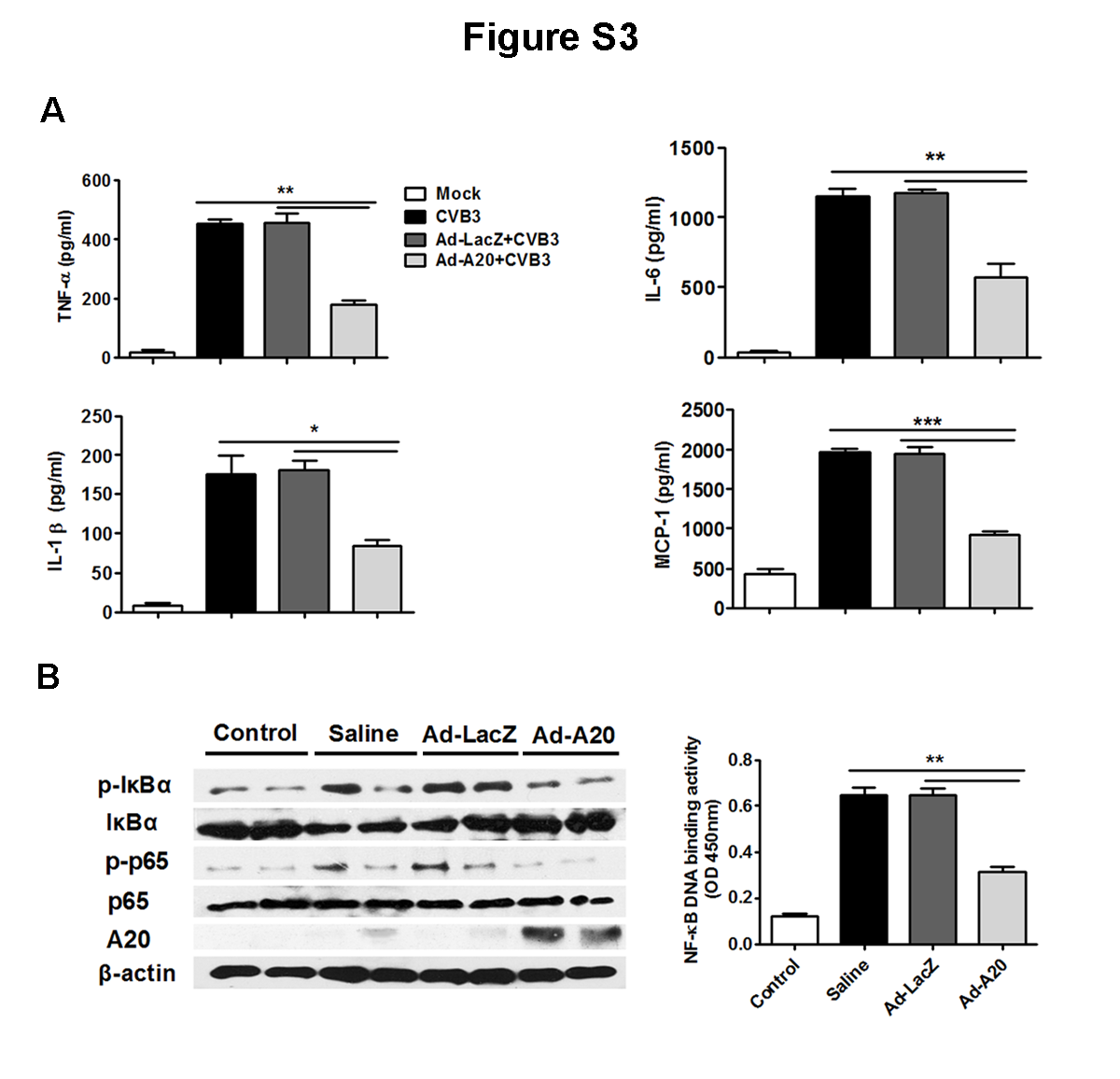

Supplement: Figure S3 — The inhibitory effect of A20 on the production of inflammatory cytokines from CVB3 infected immune cells. (A) Mouse splenocytes isolated from spleens were pre-infected with adenovirus (Ad-LacZ or Ad-A20) to over-express A20 or not. Then they were exposed to CVB3 (MOI = 10) for 24 h. The culture medium was collected and cytokines expression was assayed by ELISA. Data were presented as the means±SEM of three separate experiments. *, P<0.05; **, P<0.01; ***, P<0.001. (B) Mice were intravenously injected with saline or 3×10^9^ pfu of either Ad-A20 or Ad-LacZ 2 days before 103 TCID50 dose of CVB3 infection at day 0. Mice without infection were as control group. 7 days post-infection, splenocytes were isolated from spleens and lysed with RIPA for western blot analysis with the indicated antibodies. NF-κB DNA binding activity was analyzed by NF-κB p65 transcription factor assay kit. Data show the means±SEM of 5 mice per group. **, P<0.01. (TIF) [file pone.0046515.s003.tif]
